# Feasibility and validation of trans-valvular flow derived by four-dimensional flow cardiovascular magnetic resonance imaging in pacemaker recipients

**DOI:** 10.1016/j.mri.2020.08.024

**Published:** 2020-12

**Authors:** Christopher E.D. Saunderson, Maria F. Paton, Amrit Chowdhary, Louise A.E. Brown, John Gierula, Anshuman Sengupta, Christopher Kelly, Pei G. Chew, Arka Das, Thomas P. Craven, Rob J. van der Geest, David M. Higgins, Liang Zhong, Klaus K. Witte, John P. Greenwood, Sven Plein, Pankaj Garg, Peter P. Swoboda

**Affiliations:** a Department of Biomedical Imaging Science, Leeds Institute of Cardiovascular and Metabolic Medicine, University of Leeds, and Leeds Teaching Hospitals NHS Trust, UK; b Department of Cardiology, Leeds Teaching Hospitals NHS Trust, Leeds, UK; c Division of Image Processing, Leiden University Medical Centre, Leiden, the Netherlands; d Philips, Guildford, UK; e National Heart Research Institute Singapore, National Heart Centre Singapore, Duke-NUS Medical School, National University of Singapore, Singapore; f Academic Radiology, Infection, Immunity & Cardiovascular Disease, University of Sheffield, Sheffield, UK

## 1 Introduction

Approximately 524 cardiac pacemakers per million people are implanted in Europe per year with an increasing year-on-year trend [1]. It is estimated that up to 75% of pacemaker recipients will need magnetic resonance imaging (MRI) in their lifetime [2]. The burden of cardiovascular disease in pacemaker recipients, coupled with the increasingly prominent role of cardiac MRI in European guidelines for the diagnosis, management and monitoring of patients with cardiovascular disease has meant providing cardiac MRI to this population has become a necessity [3,4]. The advent of MRI conditional pacemakers has facilitated safe scanning of these patients although individual manufacturers' restrictions remain in place.

The feasibility and safety of performing cardiac MRI in pacemaker patients for acquisition of cines, late gadolinium imaging and perfusion has previously been established [[5], [6], [7]]. Furthermore there is increasing evidence that cardiac MRI in patients with implantable cardiac devices can often aid diagnosis or change clinical management [5,8]. Four-dimensional flow (4D flow) cardiac MRI is one of the emerging MRI techniques which has demonstrated high accuracy and precision for intracardiac flow and haemodynamic assessment [9,10]. Due to its advantages over two-dimensional phase contrast acquisition s and other Doppler based imaging methods, it is being increasingly advocated for challenging cases of congenital heart disease, valvular heart disease and haemodynamic assessment [[11], [12], [13]]. Retrospective valve tracking methods have been shown to be accurate and reliable for the assessment of valvular flow and regurgitation quantification [9,14]. However the feasibility, safety and reliability of this technique remains to be confirmed in patients with pacemakers.

We hypothesised that 4D flow cardiac MRI is feasible in patients with pacemakers and can accurately quantify valvular flow. Therefore, the main aims of the study were to (1) assess the feasibility of performing 4D flow in patients with MRI conditional pacemakers and (2) investigate the consistency and reliability of retrospective valve tracking in quantification of valvular flow in patients with pacemakers in both atrial (AOO) and dual chamber (DOO) asynchronous pacing modes.

## 2 Materials and methods

### 2.1 Study population

The study was approved by the local Ethics Committee and the study complied with the Declaration of Helsinki. All patients gave written informed consent before MRI examinations.

Thirteen patients with MRI conditional dual chamber pacemakers were prospectively recruited from a single centre. Inclusion criteria: Adults (aged over 18), MRI conditional dual chamber pacemaker system, ventricular pacing burden of less than 5% on most recent device interrogation. Exclusion criteria: Contraindication to MRI (including non-MRI conditional pacemakers, intra-orbital debris, severe claustrophobia), pregnant or breastfeeding, history of prior myocardial infarction, moderate to severe valvular heart disease and known structural heart disease.

### 2.2 Device programming

Prior to entering the MRI room, the patients underwent full pacemaker interrogation which included determination of battery voltage, lead impedance, pacing thresholds and P- and R-wave sensing amplitude. Devices were then programmed into manufacturer specific MRI safe mode. Patients were programmed to either AOO or DOO asynchronous pacing, in an arbitrary fashion, at 10 beats per minute above intrinsic heart rate to avoid competition. 12 lead electrocardiograms (ECG) were performed prior to MRI to ensure atrial pacing with intrinsic atrioventricular conduction (AOO mode) and sequential atrial and ventricular pacing (DOO mode). All patients were scanned in both AOO and DOO pacing modes during a single visit in order to evaluate feasibility of 4D flow derived valvular flow quantification in different pacing modes and the effect of the pacing mode on artefacts. Throughout the MRI examination patients were monitored using vectrocardiogram (VCG) signal and non-invasive blood pressure measurements. Following MRI a safety check was performed assessing the device battery voltage, lead impedance, pacing thresholds and sensing amplitudes and compared to values obtained prior to the MRI. Patients were then reprogrammed to pre MRI device settings.

### 2.3 Cardiovascular magnetic resonance

All patients had cardiac MRI imaging at 1.5 Tesla (Ingenia, Philips, Best, The Netherlands) with a phased array receiver coil (24-channel equipped with Philips dStream digital broadband MR architecture technology) between November 2017 and October 2018. The mean time between device implantation and MRI examination was 281 days (range: 88–853 days). All patients were scanned in normal operating mode (Upper limit of SAR level up to 2 W/kg body weight) with maximised gradient slew rate up to 200 T/m/s and according to the manufacturer's specific device instructions.

### 2.4 Image acquisition

The MRI protocol was as follows:

1. Survey images
2. Cine imaging: Acquired using balanced steady state free precession (bSSFP) in a single slice breath-hold sequence. Images obtained included a LV volume contiguous short axis stack as well as two, three and four chamber views. Typical image parameters were as follows: Slice thickness 10 mm, echo time (TE) 1.5 milliseconds (ms), repetition time (TR) 3 ms, flip angle 60°, SENSE factor 2 with 30 phases per cardiac cycle.
3. Whole heart 4D flow: Field of view (FoV) was planned in the transaxial plane with changes to FoV and number of slices performed as necessary to ensure whole heart coverage. Acquisition was performed using a fast field echo (FFE) pulse sequence [EPI based with sensitivity encoding (SENSE) acceleration, 3D] as previously described with retrospective ECG triggering [15]. Acquisition voxel size approximately 3x3x3mm. Typical scan parameters were as follows: TE 3.5 ms, TR 13 ms, flip angle 10°, velocity encoding (VENC) 150 cm/s, FoV 400 mm, number of signal averages 1, EPI acceleration factor of 5 and SENSE factor of 2. Images were acquired during free breathing with no respiratory motion correction. Number of slices was 39 with temporal resolution of 40 ms. Number of reconstructed phases was set at 30.
4. Patients were taken out of the MRI room and the device was re-programmed to alternate pacing mode at the same base rate and steps 1 to 3 were repeated.

### 2.5 Image analysis

Image analysis was performed offline using MASS software (Version 2018EXP, Leiden University Medical Centre, Leiden, The Netherlands). All images were analysed by CS (2 years' experience in advanced cardiac MRI). Endocardial contours were traced on the LV short-axis (SA) cine stack at end-diastole and end-systole, with exclusion of papillary muscles and trabeculation, to determine end-diastolic volume, end-systolic volume, stroke volume and ejection fraction for both left and right ventricles (summation of disks methodology). Epicardial contours were contoured for the left ventricle at end-diastole to calculate left ventricular mass.

For each 4D flow data set, visual quality checks on the phase contrast and magnitude images were performed by CS (2 years experience in advanced cardiac MRI) and doubled checked by PG (>5 years experience in 4D flow cardiac MRI). 4D flow phase contrast and magnitude images were visually assessed across each heart valve for the presence of the following artefacts: signal void, distortions (particularly due to the presence of pacemaker lead) and phase dispersion. The images were graded according to a 4-point scale similar to previously published work [15]. 0: excellent quality with no artefacts, 1; good quality with minimal blurring artefacts, 2; moderate quality with moderate blurring or distortion artefacts, 3; poor quality with severe artefacts in the area of interest leading to potentially non-evaluable data. Phase unwrapping was performed on source images if aliasing occurred in the region of interest according to previous guidelines on phase contrast methods [16]. Spatial misalignment of 4D flow to cine imaging was corrected prior to flow analysis. This was achieved by visualising velocity vectors in 4-chamber view in peak systole and repositioning them over the descending aorta and in 3-chamber view in peak systole and repositioning them over the ascending aorta. Similar checks were performed in diastole for peak mitral inflow velocity vectors in 2-, 3- and 4-chamber views.

**Fig. 1:**
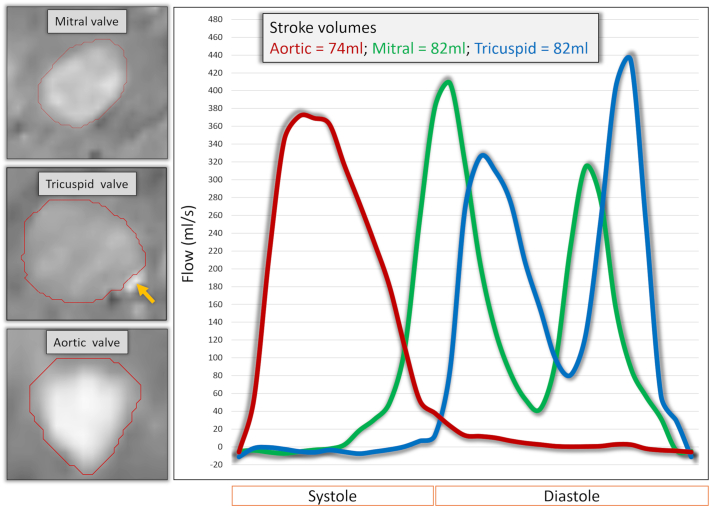
Example of segmentation of valvular flow contours on the phase contrast multiplanar reconstruction. For the tricuspid valvular flow, we just excluded the area with artefact from through plane valvular flow quantification (orange arrow). The right hand panel demonstrates flow curves for the same patient in AOO mode with comparable stroke volumes through the 3 valvular planes.

All 4D flow assessments were performed using validated retrospective valve tracking techniques with the measurement planes positioned perpendicular to inflow or outflow direction on two-, three- and four-chamber cines [14]. Background velocity correction (for correction of through plane motion and phase offset) was used from velocity sampled in the myocardium as per guidelines on phase contrast methods [16]. Contour segmentation was performed manually. Flow was determined over the entire cardiac cycle and stroke volume was calculated by the absolute forward flow minus any regurgitant flow. Susceptibility-related miscalculations of flow in certain image pixels, due to the presence of pacemaker leads within the right heart chambers, were expected to be present across the tricuspid valve on reformatted images (Supplemental fig. 1). Therefore tricuspid valve planes were manually contoured twice; initially to include the entire tricuspid orifice area and then subsequently with exclusion of miscalculated pixels that occurred because of the pacing lead (Fig. 1). To assess inter-observer variability of 4D flow derived stroke volumes a second observer (AC) repeated the analysis for all three evaluated heart valves, with exclusion of miscalculated pixels in the tricuspid valve plane, and was blinded to the previous analysis.

### 2.6 Statistical analysis

Statistical analysis was performed using SPSS 21 (International Business Machines, Armonk, New York, USA). Normality for quantitative data was established using the Shapiro-Wilk test. Continuous data measurements are presented as mean ± standard deviation. For image quality analysis the Wilcoxon signed rank test was performed to establish significant differences. For investigating agreement between left and right ventricular stroke volumes from cine imaging and aortic, mitral and tricuspid stroke volumes derived from 4D flow we used repeated measures analysis of variance (ANOVA) with Bonferroni adjustment for post-hoc analysis. Bland-Altman plots were used to both visually assess the agreement between the methods and investigate the bias (in percentage). Association between aortic and mitral and tricuspid stroke volumes was performed using Pearson correlation coefficient test. For inter-observer analysis the coefficient of variation (CV) was calculated using the root mean square method and reliability was assessed using intraclass correlation coefficient (ICC). For pre and post MRI device parameters a paired samples *t*-test was performed for normally distributed variables and the Wilcoxon signed rank test for not normally distributed variables. A *p* value <0.05 was considered significant.

## 3 Results

### 3.1 Patient characteristics

All thirteen patients, mean age 66 ± 11 years, seven males, completed the full study protocol. Five patients were assigned to an initial AOO pacing rhythm and the remainder to DOO first. A summary of the baseline demographic characteristics of the study participants and cine volumetric parameters in AOO pacing mode is provided in Table 1. The pacemaker and lead details for patients can be seen in Table 2.

**Table 1:** Baseline characteristics of patients recruited to study. Cine volume results obtained during AOO pacing mode.

Parameter	All patients (*n* = 13)
Female gender	6 (46%)
Age (yr)	66 ± 11
Heart rate (bpm)	81 ± 10
Height (cm)	170 ± 12
Weight (kg)	84 ± 20


Cine volumetric results	
LVEDV (ml)	120 ± 30
LVESV (ml)	49 ± 9
LVSV (ml)	71 ± 18
LVEF (%)	59 ± 4
LV Mass (gram)	74 ± 20
RVEDV (ml)	114 ± 28
RVESV (ml)	45 ± 12
RVSV (ml)	69 ± 17
RVEF (%)	60 ± 4

Abbreviations: LVEDV: Left ventricular end diastolic volume, LVESV: Left ventricular end systolic volume, LVSV: Left ventricular stroke volume, LVEF: Left ventricular ejection fraction, LV: Left ventricle, RVEDV: Right ventricular end diastolic volume, RVESV: Right ventricular end systolic volume, RVSV: Right ventricular stroke volume, RVEF: Right ventricular ejection fraction.

**Table 2:** Pacemaker and lead models in the study population.

Manufacturer	Model	Number
Implantable Pulse Generator		
Boston Scientific	Proponent MRI (EL231)	5
Medtronic	Ensura DR MRI (EN1DR01)	2
St Jude Medical	Assurity MRI (PM2272) Endurity MRI (PM2172)	2 4

**Lead**		
Boston Scientific	Ingevity MRI (7731, 7732, 7735, 7736, 7741, 7742)	10
Medtronic	Capsure Fix (5076)	4
St Jude Medical	Tendril STS (2088TC) Tendril MRI (LPA1200M) Isoflex (1944)	8 2 2

### 3.2 Safety and device parameters

All examinations were completed safely with no adverse clinical events and no unusual symptoms reported during the scan. All devices were interrogated before and immediately after MRI (Table 3). No significant differences were noted between battery voltage, lead impedance, capture threshold or P- and R-wave amplitude. No individual changes in lead parameters were considered clinically significant.

**Table 3:** Comparison of device parameters before and immediately after the MRI examination.

Parameter	Pre MRI value	Post MRI value	*p*-value
Pacing lead impedance (Ω) − Atrial lead − Ventricular lead	527.5 ± 94.1 665.6 ± 146.6	514.5 ± 66.9 634.8 ± 154.2	0.64 0.11
Pacing lead capture threshold (V) − Atrial lead − Ventricular lead	0.6 ± 0.2 0.9 ± 0.4	0.6 ± 0.2 0.8 ± 0.2	0.76 0.92
Battery Voltage (V) (*n* = 9)*	3.02 ± 0.1	3.02 ± 0.1	NA
P-wave amplitude (mV)	4.0 ± 1.4	4.1 ± 1.4	0.48
R-wave amplitude (mV)	12.3 ± 5.6	12.1 ± 5.3	0.95

*Boston Scientific devices were excluded as the programmer does not given a numerical value for battery voltage.

### 3.3 Image quality assessments

**Fig. 2:**
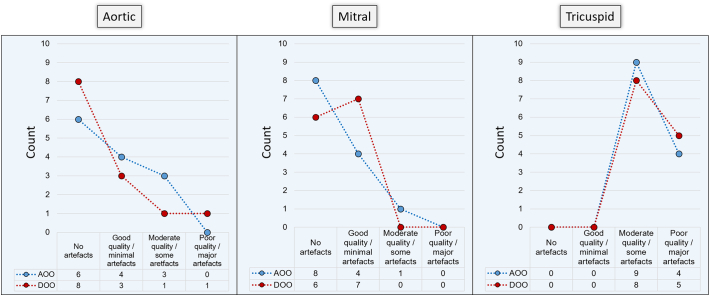
Qualitative assessment of flow in the raw data prior to valvular plane reconstruction. Even though poor quality for tricuspid flow was more often noted, by removing the miscalculated pixels, we were able to quantify tricuspid stroke volume.

Minor banding artefacts secondary to the implantable pulse generator (IPG), predominantly in apical slices, in SA cine images were observed in 5 patients in both pacing modes. Artefact scoring for phase and magnitude images across the aortic and mitral valves was similar with generally no or minimal artefacts observed in both AOO and DOO pacing modes (Fig. 2). Overall there was no significant difference in the presence of artefacts on images between pacing modes (nil; *p* = 1.0, minimal; *p* = 0.63, moderate; *p* = 0.06 or severe; *p* = 0.18). However, due to the presence of the pacing leads, moderate or severe artefacts, due to susceptibility-related miscalculations of flow, were seen on phase images across the tricuspid valve in all patients (Supplemental fig. 1).

### 3.4 Tricuspid flow quantification - with/without inclusion of pacemaker lead artefact

On direct comparison of tricuspid flow with the inclusion of the RV lead artefact versus exclusion of the lead artefact, we noted that when we included the RV lead artefact there was significant overestimation of transvalvular stroke volume (SV) in both AOO (77 ± 18mls vs 69 ± 18mls; *p* < 0.001) and DOO modes (74 ± 17mls vs 68 ± 17mls; p < 0.001). Therefore the values that excluded the RV lead artefact were used for subsequent comparison with stroke volumes of left sided heart valves (Fig. 1). No significant tricuspid regurgitation was observed, after exclusion of RV pacing lead artefact, with negligible negative flow seen in either AOO or DOO pacing modes (1.43 ± 1.36 mls vs. 1.91 ± 0.93 mls respectively; *p* = 0.26).

### 3.5 Consistency of 4D flow derived flow volume assessment

**Fig. 3:**
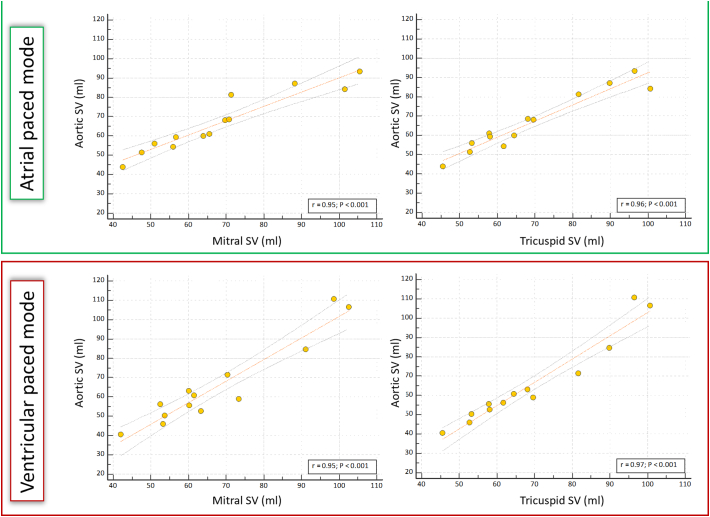
Scatter plots of aortic stroke volume (SV) against mitral and tricuspid SV for AOO and DOO pacing modes to investigate consistency between methods. Excellent correlation was noted for all (r>0.95).

**Fig. 4:**
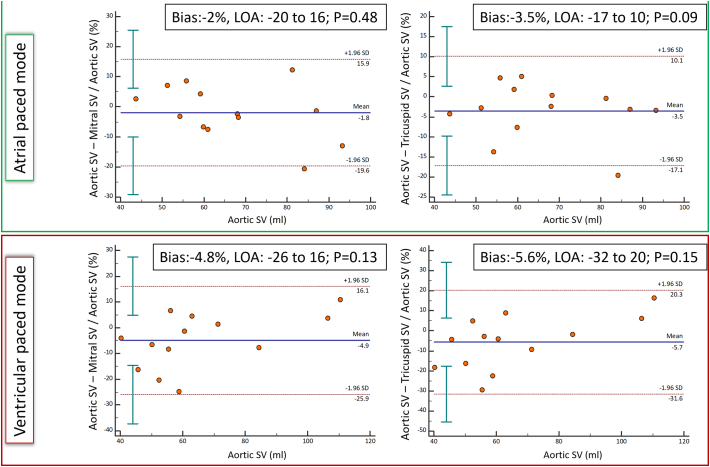
Bland-Altman analysis for the assessment of aortic stroke volume (SV) against mitral and tricuspid SV for AOO and DOO pacing modes. No significant differences was noted on Bland-Altman analysis.

In AOO pacing mode SV for the aortic valve correlated with both mitral (*r* = 0.95; *p* < 0.001) and tricuspid (*r* = 0.96; p < 0.001) valvular SVs (Fig. 3). Bias for SV in AOO pacing mode was highest between the aortic and tricuspid valves (−3.5%, LOA −17 to 10%; *p* = 0.09) although was not significant (Fig. 4). In DOO pacing mode, SV for the aortic valve correlated with both mitral (r = 0.95; p < 0.001) and tricuspid (*r* = 0.97; p < 0.001) valvular SVs (Fig. 3). No significant bias for the SV in this pacing mode was observed between aortic valve and mitral and tricuspid valves (−4.8%, LOA −26 to 16%; *p* = 0.13 and − 5.6%, LOA −32 to 20%; *p* = 0.15 respectively) (Fig. 4). No significant aortic or mitral regurgitation was seen on reformatted images. There was negligible and non-significant negative flow between AOO and DOO pacing modes across both the aortic (AOO: 0.39 ± 0.87 mls vs. DOO: 0.79 ± 1.24 mls; *p* = 0.23) and mitral (AOO: 0.64 ± 0.44 mls vs. DOO: 0.76 ± 0.33 mls; *p* = 0.44) valves.

### 3.6 Comparison of cine and 4D flow derived valvular stroke volumes

**Fig. 5:**
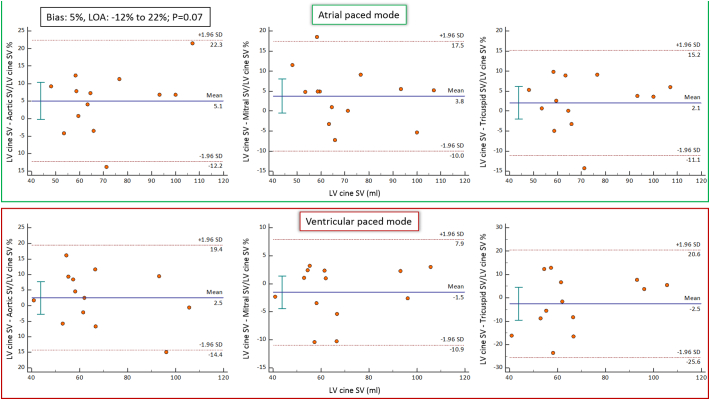
Comparison of LV short-axis cine and 4D flow derived stroke volumes (SV). Bland-Altman plots were used to investigate any significant bias between cine SV and 4D flow derived SV. The Bland-Altman analysis did not demonstrate any significant bias between cine SV and the 4D flow methods derived SV (P>0.05).

In both AOO and DOO pacing modes there was no significant difference between the mean SV obtained from short-axis cine imaging for either the left or right ventricle and 4D flow derived aortic, mitral or tricuspid SV (*p* > 0.05) (Table 4). Bland-Altman analysis did not demonstrate any significant bias between the left ventricular cine SV and 4D flow methods for each valvular SV in either pacing mode (p > 0.05) (Fig. 5).

**Table 4:** Comparison of mean stroke volume by cine and aortic/mitral/tricuspid valves derived from 4D flow according to assigned pacing mode. No significant differences were noted.

	Stroke Volume (ml)					p-value⁎
	LV cine	RV cine	Aortic	Mitral	Tricuspid	
Atrial pacing mode (AOO)	71 ± 18	69 ± 17	67 ± 15	69 ± 20	69 ± 18	0.15
Ventricular pacing mode (DOO)	67 ± 19	68 ± 19	66 ± 22	68 ± 19	68 ± 17	0.70

Abbreviations: LV: Left ventricle, RV: Right ventricle.

⁎ P-value – from repeated measures ANOVA using Bonferroni post-hoc analysis.

### 3.7 Inter-observer repeatability

For the aortic and mitral valves in both pacing modes the ICC were strong with a low CV suggesting good inter-observer agreement (Table 5). For the tricuspid valve, in both pacing modes, ICC was lower with a higher CV suggesting a more modest inter-observer agreement.

**Table 5:** Inter-observer reproducibility for 4D flow derived valvular stroke volumes for both pacing modes.

	CV (%)	ICC
Atrial pacing mode (AOO)		
Aortic SV	8.7	0.912
Mitral SV	8.1	0.965
Tricuspid SV	14.0	0.762

Ventricular pacing mode (DOO)		
Aortic SV	7.6	0.921
Mitral SV	8.8	0.911
Tricuspid SV	10.2	0.861

Abbreviations: CV: coefficient of variation, ICC: intraclass correlation coefficient, SV: stroke volume.

## 4 Discussion

The present study investigated the feasibility and consistency of 4D flow derived valvular flow assessment in patients with MRI conditional pacemakers. The study demonstrates that: (1) 4D flow cardiac MRI is feasible in patients with MRI conditional pacemakers in two different pacing modes; (2) Flow across left sided (aortic and mitral) heart valves is consistent in both AOO and DOO pacing modes; (3) 4D flow derived valvular stroke volume quantification is comparable with the cine derived stroke volume; (4) Susceptibility artefacts are commonly present on the tricuspid valve plane due to the RV pacing lead but can be circumvented to some extent by excluding miscalculated pixels in close proximity to the lead.

### 4.1 Safety

All the patients in the study underwent the full protocol with no significant changes in device parameters noted between the pre and post MRI device interrogation. Therefore the current study suggests that 4D flow cardiac MRI seems not to pose any additional risk in patients with MRI conditional pacemakers if scanned in normal operating mode (SAR level up to 2 W/kg body weight) with a maximised gradient slew rate up to 200 T/m/s. These findings are in keeping with the previous literature demonstrating the safety of performing cardiac MRI on patients with MRI conditional pacemakers [6,8].

### 4.2 Image quality and qualitative assessment of flow

Imaging artefacts in patients with pacemakers occur predominantly due to the presence of ferromagnetic material within the IPG and pacing leads. However alterations of patient positioning within the scanner when changing pacing modes and movement of the pacing lead throughout the cardiac cycle could potentially further effect image quality.

On cine imaging artefact was predominantly due to the presence of IPG leading to minor banding artefacts, predominantly in the apical LV segments [6]. These artefacts were consistent between pacing modes and endocardial/blood pool definition was adequate to allow determination of stroke volume for both ventricles. The lower ferromagnetic content in pacing leads meant little to no artefact was seen on cine imaging. No visual change in artefact was observed between pacing modes.

**Fig. 6:**
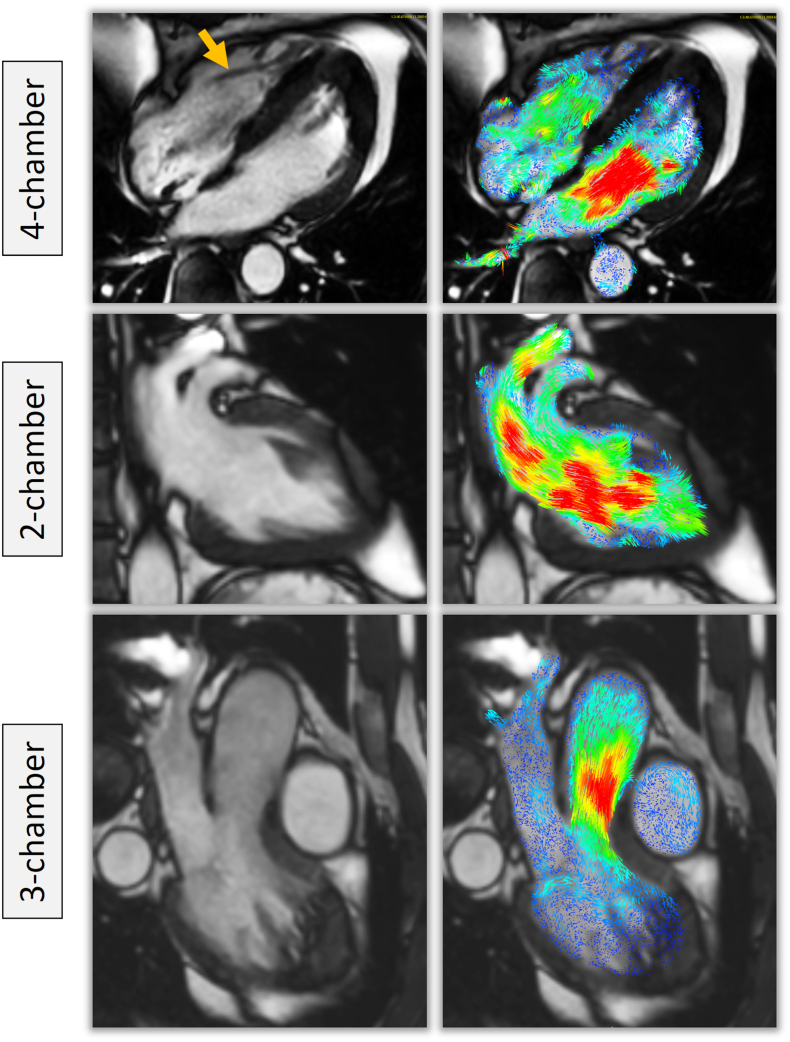
A case example demonstrating two dimensional velocity vectors superimposed over cine images in a patient with a pacemaker and right ventricular pacing lead (orange arrow). No significant artefacts were noted.

The presence of an MRI conditional pacemaker has previously been shown not to affect the image quality or generation of flow curves in 2D aortic phase contrast imaging [6]. The current study demonstrated the image quality of the phase contrast and magnitude images for 4D flow acquisition in patients with pacemakers was generally good, particularly for the left heart. The reconstructed aortic and mitral valve planes generally had little or no artefact which allowed robust quantification of valvular flow. Furthermore, no significant artefacts were noted in velocity vector visualisation on cine images for either the left or right heart (Fig. 6).

Miscalculated pixels secondary to the susceptibility generated by the RV pacing lead were consistently seen on the phase and magnitude 4D flow data of the tricuspid valve plane. These were generally limited to a few pixels in close proximity to the RV lead. Contouring the entire orifice area, including the miscalculated pixels, led to overestimation of stroke volume relative to the left sided heart valves. In all of our cases the RV pacing lead was at the edge of the valve orifice area and therefore repeat manual contouring with exclusion of the miscalculated pixels created by the pacing lead meant stroke volumes comparable to the aortic and mitral valves could be determined. This technique clearly requires additional post processing time and the effect of flow measurements when the pacing lead is positioned in the middle of the valve orifice is unknown. However, in the latter circumstance we would suggest a second contour be drawn around the artefact and this value deducted from the total stroke volume for the entire orifice area. There was no significant difference in artefacts between the pacing modes.

### 4.3 Quantitative assessment of transvalvular stroke volume

Current methods of quantifying valvular flow and intra-cardiac shunts are based on Doppler echocardiography techniques that are often limited by acoustic windows, difficulties with velocity assessment due to beam alignment and are therefore often dependent on operator experience meaning measurements often have limited reproducibility [[17], [18], [19]]. Over recent years 4D flow derived measurements using valvular stroke volumes obtained by the retrospective valve tracking techniques have been shown to be accurate, consistent and reproducible across all four heart valves [9,14,20,21]. The present study has shown that stroke volume quantification, particularly for the left sided heart valves, by retrospective valve tracking is consistent in patients with pacemakers and is reproducible in two separate pacing modes. These findings are consistent with a previous study by Garg et al. which used similar undersampling methods for faster 4D flow whole-heart acquisitions - EPI acceleration with a factor of five and a SENSE factor of 2 [15]. This study showed robust correlation between aortic and mitral net forward flow (*r* = 0.94) in healthy volunteers which is comparable to our results in both pacing modes (*r* ≥ 0.95). Importantly inter-observer repeatability was less robust for the tricuspid valve, which may be a consequence of the artefact generated by the RV pacing lead, and further work is needed to determine the effect of the pacing lead on these 4D flow derived stroke volumes. 4D flow derived valvular stroke volumes were also consistent with stroke volumes determined by cine imaging.

### 4.4 Clinical applications

The demonstration of feasibility as well as the consistency of 4D flow derived flow measurements is important as the number of pacemaker implantations in Europe is on an upward trend due to the ageing population [1]. Given the burden of cardiovascular disease in pacemaker recipients it seems probable that a significant proportion of them will require cardiac MRI during their lifespan given cardiac MRI is often recommended in International guidelines [3,4]. Cardiac MRI has already been shown to provide important diagnostic and management changing information in patients with pacemakers [5,8]. 4D flow MRI can play a vital additive role as it provides accurate and consistent intra-scan assessment of blood flow with strong rescan reproducibility. Indeed 4D flow allows sampling and quantification of blood flow in any direction within the 3D volume so may forgo the need for a series of 2D cine breath held phase contrast sequences and retrospective valve tracking techniques may improve assessment of transvalvular flow [9,22]. This may be particularly pertinent in the repeated imaging of pacemaker patients with congenital or valvular heart disease where serial assessment of regurgitant volumes or shunts is required [10,[23], [24], [25]].

### 4.5 Possible future applications

Right ventricular apical pacing induces electrical and mechanical dyssynchrony leading to alterations in cardiac haemodynamics and can lead to adverse cardiac remodelling and even the development of heart failure in the long-term [26,27]. The mechanisms underpinning the development of this so called ‘pacing induced cardiomyopathy’ however are incompletely understood. 4D flow cardiac MRI affords the evaluation of a series of advanced cardiac haemodynamic parameters such as kinetic energy (KE), turbulent KE, particle tracing and vortex visualisation [22]. These parameters are predominantly research tools but have been suggested as subclinical markers of LV dysfunction with reductions in average LV KE and end diastolic KE observed in patients with ischaemic heart disease and little or no LV dysfunction [28,29]. More recently it has been shown in heart failure patients with dyssynchrony from left bundle branch block (LBBB) that LV filling forces are more orthogonal to main LV flow direction during early diastole and the direct flow entering the LV has lower KE when compared to those without LBBB [30,31]. Suwa et al. have also demonstrated changes in vortex size and core locations during diastole in patients with heart failure suggesting vortex formation plays a role in LV ejection and filling [32]. Therefore these metrics may allow us to evaluate how flow haemodynamics change in pacing induced dyssynchrony and may contribute to the pathophysiology of pacing induced left ventricular dysfunction and development of heart failure. Indeed recent work using echocardiographic particle image velocimetry has demonstrated that blood flow momentum and KE dissipation are altered with RV apical pacing and associated with deterioration in global longitudinal strain, highlighting the potential role that altered flow dynamics may play in adverse cardiac remodelling over the longer term in these patients [33]. Although full evaluation of intra-cardiac flows in patients with pacemakers would require manufacturers to allow greater flexibility in device programming within the MRI environment.

### 4.6 Limitations

There were several limitations to our study. The number of patients recruited to this study remains small and the implanted pacemakers were from a limited number of manufacturers with MRI conditional models. This study did not evaluate pulmonary valvular flow as the relevant right ventricular outflow tract cines for retrospective valve tracking planning were not acquired. The artefact created by the RV pacing lead meant tricuspid stroke volume was overestimated. Although excluding these miscalculated pixels meant that stroke volumes were consistent with aortic and mitral valves this could have important implications for calculating regurgitant volumes across the tricuspid valve, particularly if this occurs in close proximity to the pacing lead. The 4D flow sequence used in this study was not respiratory navigated. However respiratory navigated sequences have a longer acquisition time and this may preclude their application in clinical workflows. Furthermore in healthy volunteers the use of respiratory motion compensation has been shown to have no significant effect on intra-cardiac flow quantification [34]. This study did not recruit patients with significant valvular heart disease, especially patients with tricuspid regurgitation. Future studies will need to establish the reliability of 4D flow in quantifying pulmonary and tricuspid flow in pacemaker patients particularly as inclusion of miscalculated pixels due to the RV pacing lead may augment derived stroke volumes. This is not as relevant for the left heart as the artefacts are minimal. Larger studies are required to fully evaluate safety of 4D flow cardiac MRI across a wider range of devices including cardiac resynchronisation pacemakers and implanted cardioverter-defibrillators.

## 5 Conclusion

Whole-heart, 4D flow cardiac MRI in patients with MRI conditional pacemakers is feasible. Retrospective valve tracking techniques allow assessment of stroke volumes, particularly across left sided heart valves, irrespective of pacing mode and are comparable to stroke volumes obtained using cine imaging. Further research is needed in patients with defibrillators and cardiac resynchronisation devices to evaluate whether better device optimisation is possible by 4D flow guided cardiac haemodynamics.

## Grant support

AD is funded by 10.13039/501100000327Heart Research UK, United Kingdom (RG2668/18/20). ED is supported by a grant from the 10.13039/501100000274British Heart Foundation, United Kingdom (FS/13/71/30378). SP is supported by a grant from the 10.13039/501100000274British Heart Foundation, United Kingdom (CH/16/2/32089).

## Disclosures

The authors have reported that they have no relationships relevant to the contents of this paper to disclose.

## Appendix A Supplementary data

Supplemental figure 1 Phase contrast and reformatted images of the tricuspid valve in a patient with a pacemaker. Image 1

## References

[1] Raatikainen M.J.P., Arnar D.O., Merkely B. (2017). A decade of information on the use of cardiac implantable electronic devices and interventional electrophysiological procedures in the European Society of Cardiology Countries: 2017 report from the European Heart Rhythm Association. Europace..

[2] Brignole M., Auricchio A., Baron-Esquivias G. (2013). 2013 ESC guidelines on cardiac pacing and cardiac resynchronization therapy: the Task Force on cardiac pacing and resynchronization therapy of the European Society of Cardiology (ESC). Developed in collaboration with the European Heart Rhythm Association (EHRA). Eur Heart J.

[3] von Knobelsdorff-Brenkenhoff F., Schulz-Menger J. (2016). Role of cardiovascular magnetic resonance in the guidelines of the European Society of Cardiology. J Cardiovasc Magn Reson.

[4] Ebert M., Jander N., Minners J. (2016). Long-term impact of right ventricular pacing on left ventricular systolic function in pacemaker recipients with preserved ejection fraction: results from a large single-Center registry. J Am Heart Assoc.

[5] Bhuva A.N., Kellman P., Graham A. (2019). Clinical impact of cardiovascular magnetic resonance with optimized myocardial scar detection in patients with cardiac implantable devices. Int J Cardiol.

[6] Klein-Wiele O., Garmer M., Busch M. (2017). Cardiovascular magnetic resonance in patients with magnetic resonance conditional pacemaker systems at 1.5 T: influence of pacemaker related artifacts on image quality including first pass perfusion, aortic and mitral valve assessment, flow measurement, short tau inversion recovery and T1-weighted imaging. Int J Cardiovasc Imaging.

[7] Naehle C.P., Kreuz J., Strach K. (2011). Safety, feasibility, and diagnostic value of cardiac magnetic resonance imaging in patients with cardiac pacemakers and implantable cardioverters/defibrillators at 1.5 T. Am Heart J.

[8] Raphael C.E., Vassiliou V., Alpendurada F. (2016). Clinical value of cardiovascular magnetic resonance in patients with MR-conditional pacemakers. Eur Heart J Cardiovasc Imaging.

[9] Westenberg J.J., Roes S.D., Ajmone Marsan N. (2008). Mitral valve and tricuspid valve blood flow: accurate quantification with 3D velocity-encoded MR imaging with retrospective valve tracking. Radiology..

[10] Feneis J.F., Kyubwa E., Atianzar K. (2018). 4D flow MRI quantification of mitral and tricuspid regurgitation: reproducibility and consistency relative to conventional MRI. J Magn Reson Imaging.

[11] Zhong L., Schrauben E.M., Garcia J. (2019). Intracardiac 4D flow MRI in congenital heart disease: recommendations on behalf of the ISMRM Flow & Motion Study Group. J Magn Reson Imaging.

[12] Barker N., Fidock B., Johns C.S. (2019). A systematic review of right ventricular diastolic assessment by 4D flow CMR. Biomed Res Int.

[13] Fidock B., Barker N., Balasubramanian N. (2019). A systematic review of 4D-flow MRI derived mitral regurgitation quantification methods. Front Cardiovasc Med.

[14] Roes S.D., Hammer S., van der Geest R.J. (2009). Flow assessment through four heart valves simultaneously using 3-dimensional 3-directional velocity-encoded magnetic resonance imaging with retrospective valve tracking in healthy volunteers and patients with valvular regurgitation. Invest Radiol.

[15] Garg P., Westenberg J.J.M., van den Boogaard P.J. (2018). Comparison of fast acquisition strategies in whole-heart four-dimensional flow cardiac MR: two-center, 1.5 Tesla, phantom and in vivo validation study. J Magn Reson Imaging.

[16] Lotz J., Meier C., Leppert A., Galanski M. (2002). Cardiovascular flow measurement with phase-contrast MR imaging: basic facts and implementation. Radiographics..

[17] Galderisi M., Henein M.Y., D’Hooge J. (2011). Recommendations of the European Association of Echocardiography: how to use echo-Doppler in clinical trials: different modalities for different purposes. Eur J Echocardiogr.

[18] Lancellotti P., Tribouilloy C., Hagendorff A. (2013). Recommendations for the echocardiographic assessment of native valvular regurgitation: an executive summary from the European Association of Cardiovascular Imaging. Eur Heart J Cardiovasc Imaging.

[19] Biner S., Rafique A., Rafii F. (2010). Reproducibility of proximal isovelocity surface area, vena contracta, and regurgitant jet area for assessment of mitral regurgitation severity. JACC Cardiovasc Imaging.

[20] Kamphuis V.P., Roest A.A.W., Ajmone Marsan N. (2019). Automated cardiac valve tracking for flow quantification with four-dimensional flow MRI. Radiology..

[21] Crandon S., Elbaz M.S.M., Westenberg J.J.M. (2017). Clinical applications of intra-cardiac four-dimensional flow cardiovascular magnetic resonance: a systematic review. Int J Cardiol.

[22] Dyverfeldt P., Bissell M., Barker A.J. (2015). 4D flow cardiovascular magnetic resonance consensus statement. J Cardiovasc Magn Reson.

[23] Kamphuis V.P., van der Palen R.L.F., de Koning P.J.H. (2018). In-scan and scan-rescan assessment of LV in- and outflow volumes by 4D flow MRI versus 2D planimetry. J Magn Reson Imaging.

[24] Chelu R.G., Horowitz M., Sucha D. (2019). Evaluation of atrial septal defects with 4D flow MRI-multilevel and inter-reader reproducibility for quantification of shunt severity. MAGMA..

[25] Hsiao A., Tariq U., Alley M.T., Lustig M., Vasanawala S.S. (2015). Inlet and outlet valve flow and regurgitant volume may be directly and reliably quantified with accelerated, volumetric phase-contrast MRI. J Magn Reson Imaging.

[26] Tops L.F., Schalij M.J., Bax J.J. (2009). The effects of right ventricular apical pacing on ventricular function and dyssynchrony implications for therapy. J Am Coll Cardiol.

[27] Lamas G.A., Lee K.L., Sweeney M.O. (2002). Ventricular pacing or dual-chamber pacing for sinus-node dysfunction. N Engl J Med.

[28] Garg P., Crandon S., Swoboda P.P. (2018). Left ventricular blood flow kinetic energy after myocardial infarction - insights from 4D flow cardiovascular magnetic resonance. J Cardiovasc Magn Reson.

[29] Svalbring E., Fredriksson A., Eriksson J. (2016). Altered diastolic flow patterns and kinetic energy in subtle left ventricular Remodeling and dysfunction detected by 4D flow MRI. PLoS One.

[30] Eriksson J., Zajac J., Alehagen U. (2017). Left ventricular hemodynamic forces as a marker of mechanical dyssynchrony in heart failure patients with left bundle branch block. Sci Rep.

[31] Zajac J., Eriksson J., Alehagen U. (2018). Mechanical dyssynchrony alters left ventricular flow energetics in failing hearts with LBBB: a 4D flow CMR pilot study. Int J Cardiovasc Imaging.

[32] Suwa K., Saitoh T., Takehara Y. (2016). Intra-left ventricular flow dynamics in patients with preserved and impaired left ventricular function: analysis with 3D cine phase contrast MRI (4D-Flow). J Magn Reson Imaging.

[33] Bianco F., Cicchitti V., Bucciarelli V. (2019). Intraventricular flow patterns during right ventricular apical pacing. Open Heart.

[34] Kanski M., Toger J., Steding-Ehrenborg K. (2015). Whole-heart four-dimensional flow can be acquired with preserved quality without respiratory gating, facilitating clinical use: a head-to-head comparison. BMC Med Imaging.

