# Supplementary material for: Feasibility and validation of trans-valvular flow derived by four-dimensional flow cardiovascular magnetic resonance imaging in pacemaker recipients
Source: Magn Reson Imaging. 2020 Dec;74:46–55. doi: 10.1016/j.mri.2020.08.024 (PMC7674584; doi:10.1016/j.mri.2020.08.024)
Supplement: Supplemental figure 1 — Phase contrast and reformatted images of the tricuspid valve in a patient with a pacemaker. [file mmc1.docx]

Supplementary Material


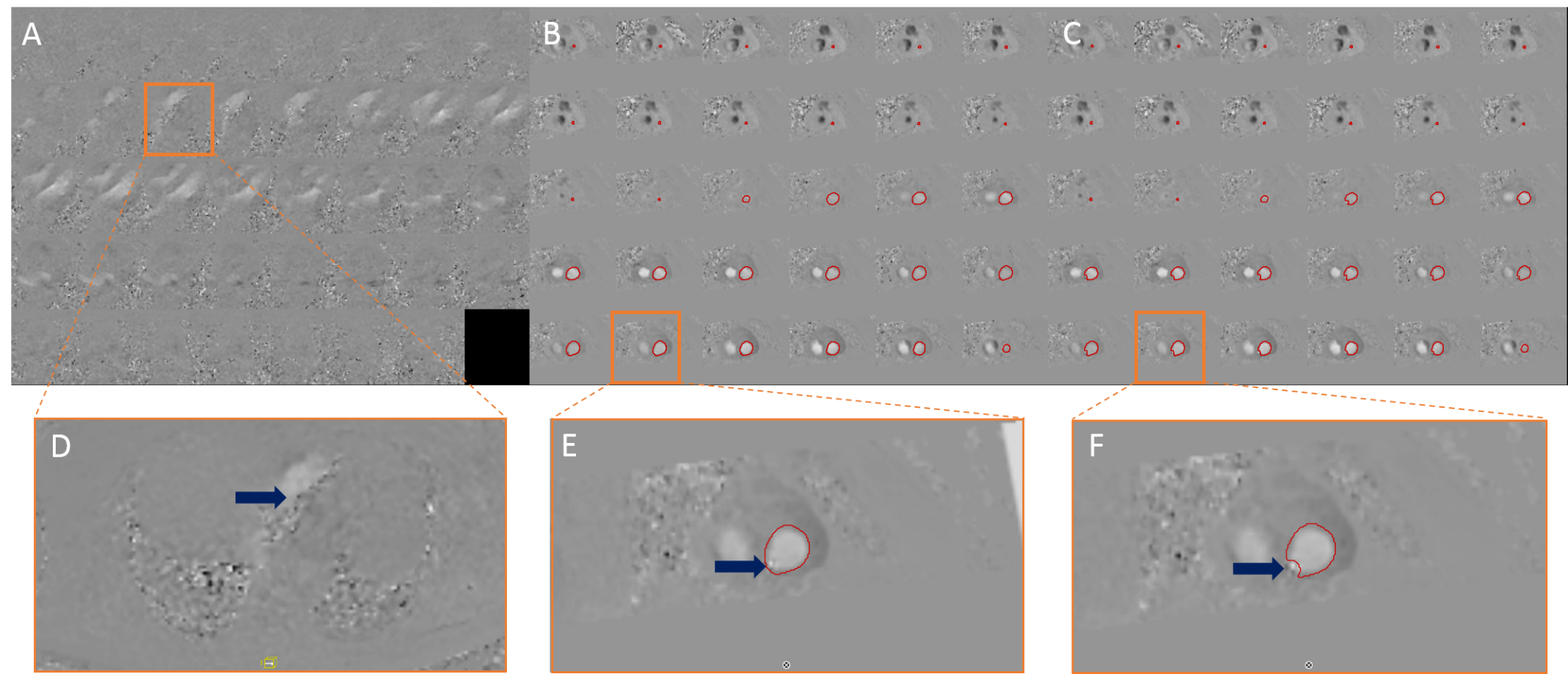


Supplemental figure 1. Phase and reformatted images of the tricuspid valve in a patient with a pacemaker.

Phase contrast images in the anterior-posterior direction demonstrate mis-calculated pixels (blue arrows) from the pacing leads in the inferior aspect of the right heart (Panels A & D). Reformatted images of the tricuspid valve plane demonstrate the presence of the mis-calculated pixels on the edge of the tricuspid valve (Panels B, C, E and F). Manual contours of the tricuspid valve with inclusion (Panel E) and exclusion (Panel F) of the mis-calculated pixels are shown.
